# Single immersion in cold water below 4 °C: A health hazard in young healthy men?

**DOI:** 10.1371/journal.pone.0324502

**Published:** 2025-05-23

**Authors:** Aneta Teległów, Iwona Cicha

**Affiliations:** 1 Department of Health Promotion, Institute of Basic Sciences, Faculty of Motor Rehabilitation, University of Physical Culture in Krakow, Krakow, Poland; 2 Cardiovascular Nanomedicine Unit, Section of Experimental Oncology and Nanomedicine (SEON), Department of Otorhinolaryngology, Head and Neck Surgery, Universitätsklinikum Erlangen, Friedrich-Alexander Universität Erlangen-Nürnberg, Erlangen, Germany; Southwest Petroleum University, CHINA

## Abstract

The circulatory system plays a significant role in the adaptation of the human body to varying environmental conditions and stress factors, such as cold water immersion. The aim of the study was to determine whether a single immersion in cold water below 4 °C, at air temperature of -15 °C constitutes a health risk in young healthy men. For this purpose, the following parameters were determined in the blood samples collected in 13 young males before and after cold water immersion: electrolytes, renal profile, liver profile, lipid profile, glucose, testosterone, thyroid-stimulating hormone, cortisol. After the immersion, a statistically significant decrease was found for Cl^–^ (p ≤ 0.027), mean values of renal profile indicators: urea (p = 0.0019), creatinine (p = 0.0007), and uric acid (p = 0.0293), as well as testosterone (p = 0.000037). In turn, higher values of estimated glomerular filtration rate (p = 0.0425), total bilirubin (p = 0.0033), aspartate transaminase (p = 0.0023), alanine transaminase (p = 0.0053), lactate dehydrogenase (p = 0.0336), creatine kinase (p = 0.0117), CK-MB isoenzyme of creatine kinase (p = 0.0028), and high-density lipoprotein (p = 0.0270) were measured after cold water immersion. No significant changes in other biochemical parameters were observed. Single cold water immersion thus resulted in significant changes of blood parameters within non-pathological limits.

## Introduction

The circulatory system plays a significant role in the adaptation of the human body to varying environmental conditions. Cold water immersion (CWI) is a stress factor [1–4], which causes heat loss and threatens homeostasis. Problems arising from short-term CWI are mainly related to physical exhaustion triggered by neuromuscular cooling [5]. In an individual at rest, even moderately cold water can lead to hypothermia. The skin, subcutaneous adipose tissue, and skeletal muscles, when at rest (in which case there is limited blood flow in them), provide thermal insulation for the organs, but cold stress constitutes an important risk factor for cardiovascular disorders [6].

Apart from the exposure time and temperature, the body response to CWI may largely depend on such individual characteristics as the specific metabolic level and the amount of subcutaneous adipose tissue. The latter is an important insulating factor owing to its low thermal conductivity and poor vascularization. One millimetre of adipose tissue thickness helps withstand cooling of 1–2 °C [7]. According to Noakes [8], the physiological response of the body exposed to cold water is variable. Tipton [9] divides it into four categories: initial response to immersion (0–3 minutes), response to short-term immersion (3–15 minutes), response to long-term immersion (> 30 minutes), and post-immersion response. In an aquatic environment with a temperature of 5 °C, the rectal temperature decreases by 1.2 °C per 20 minutes at rest, and by 1.8 °C per 20 minutes while swimming [10].

CWI was reported to induce functional changes, mainly in the cardiovascular system, through increased stimulation of the autonomic system and endocrine function modifications under conditions of enhanced heat conduction in the aquatic environment [11,12]. Janský et al. [11] noted that the main mechanism regulating cardiovascular activity under CWI conditions was an increase in blood norepinephrine concentration and raised sympathetic nervous system activity under the influence of body cooling. These authors also reported significant increases in systolic and diastolic blood pressure, with no significant changes in heart rate after 1 hour of immersion. In contrast, a study of Gabrielsen et al. showed a reduction in heart rate with a simultaneous increase in cardiac stroke volume and blood pressure [13]. Under the conditions of enhanced heat loss to the environment, an increase is observed in the secretion of hormones that stimulate metabolism. Multiple studies reported that body exposure to cold water enhances the activity of the sympathetic-adrenal system [14,15], as well as the secretion of norepinephrine [16–18], adrenaline, and cortisol [14,15]. Norepinephrine influences thermoregulation (metabolic heat production) via β-adrenergic receptors in skeletal muscles [16] and its levels can increase to 180% of baseline value within 2 minutes of immersion in 10 °C water [17]. Šrámek et al. [18] observed an increase in concentrations of norepinephrine by as much as 530% in individuals immersed in water at a temperature of 14 °C for 1 h. An increase in the level of catecholamines circulating in the blood also enhances cellular metabolism, which is referred to as non-shivering thermogenesis and increases heat production by 10–15% [19]. Impulses from the thermoregulatory centre cause strong activation of the sympathetic nervous system, which results in the stimulation of α-adrenergic receptors and a pronounced contraction of skin vessels, preventing heat transfer from the body interior to the skin and further heat loss to the environment via the abovementioned physical mechanisms. Only the heat that has passed through the insulator constituted by the subcutaneous adipose tissue is transferred to the environment.

According to Petersen and Fyfe [20], post-exercise CWI is a commonly used method to help athletes recover. However, the literature on the results of repeated CWI on adaptation to exercise, particularly resistance exercise, is scarce. In spite of the fact that post-exercise CWI may improve short-term recovery after resistance exercise, it has been reported that CWI exerts almost no effect on physiological adaptation to resistance training; this also refers to muscle hypertrophy. In the context of training-recovery balance, much attention is being paid to repeated CWI as a means to enhance recovery and training quality [21]. Post-training CWI at 15 °C which lasts 5 minutes can prevent or diminish the decrease in parasympathetic activity commonly observed after exercise and is furthermore related to better sleep quality [22].

Our previous study, which reported haemorheological response of the human body to immersion in 4 °C water and in vitro effects of low temperature on the primary cells of human vascular endothelium [23], demonstrated that moderate hypothermia can worsen erythrocyte deformability parameters and strongly activates endothelial cells in vitro. The main objective of the present study was to verify if the immersion in water at a temperature below 4 °C and air temperature of -15 °C constitutes a health hazard in young males. For this purpose, the following blood parameters were determined before and after CWI: electrolytes, renal profile, liver profile, lipid profile, glucose, testosterone, thyroid-stimulating hormone (TSH), cortisol. The observed significant changes in blood parameters indicated a strong response to cold-induced stress, but were within non-pathological limits.

## Materials and methods

### Participants

The subjects of the study were 13 healthy male physiotherapy students at the Faculty of Physical Rehabilitation, University of Physical Education in Krakow, aged 21–25 years. None of them practised winter swimming. Each participant received a physician’s consent to be involved in the study and underwent physiotherapist consultation. The exclusion criteria involved any chronic disease such as diabetes, heart disease, metabolic disorder, or endocrine disorder, as well as smoking, active infections, and neoplasms. The individuals became fully acquainted with the details of the study and provided their written consent to take part. 03.10.22 of the start and 21.10.22 end of the recruitment period for this study.The research procedures followed the Declaration of Helsinki with its amendments [23].

### Experimental set-up

The participants were immersed for 2–3 minutes in a tub of cold water, made of weather- and water-resistant, thermally modified wood and containing water at a temperature below 4 °C. The tub was positioned in a refrigerator truck and the air temperature was maintained at –15 °C. The total time of cold exposure was approximately 5 min. Before CWI, the study subjects were trained by multiple Guinness World Record holder Valerjan Romanovski, who also supervised the immersion. Before and immediately after CWI, the participants’ blood pressure, heart rate, and body temperature were determined.

Before and within 7 minutes after CWI, a qualified nurse collected 5 ml of the subjects’ fasting blood from an ulnar vein in the Blood Physiology Laboratory of the Central Research and Development Laboratory, University of Physical Education in Krakow. The collected blood was placed in Vacuette EDTA K2 tubes and in clot activator tubes. The biochemical blood indicators were assessed on the day of blood collection in the abovementioned laboratory, in the Department of Clinical Analytics and Biochemistry of Maria Sklodowska-Curie National Research Institute of Oncology, regional branch in Krakow, and in the Diagnostyka S.A. laboratory in Krakow, Poland. One study subject was excluded from analyses due to failure of collecting a sufficient volume of blood after CWI because of strong vasoconstriction.

A Cobas device (Roche, Mannheim, Germany) was applied in the study with an ion-selective electrode module to quantify potassium, sodium, and chloride ions. The sodium and potassium electrodes are based on natural carriers, and the chloride electrode is based on an ion exchanger. In the present study, the concentration of the following electrolytes was analysed: sodium ions (Na^+^) [mmol/l], potassium ions (K^+^) [mmol/l], and chloride ions (Cl^–^) [mmol/l].

The renal and liver profiles were determined with the use of a Roche/Hitachi Cobas c 311 analyser (Roche Diagnostics, Basel, Switzerland), which automatically calculates the analytical activity of each substance. The colorimetric method and reagent kits were employed. For the renal profile, urea [mmol/l], creatinine [mmol/l], uric acid [μmol/l], and estimated glomerular filtration rate (eGFR) [ml/min/1.73 m^2^] were measured. In the case of the liver profile, total bilirubin [μmol/l], aspartate transaminase (AST) [U/l], alanine transaminase (ALT) [U/l], gamma-glutamyltransferase (GGT) [U/l], and alkaline phosphatase [U/l] were evaluated. The following blood biochemical indices were measured in plasma by using a biochemical analyzer Roche/Hitachi (Cobas c 501, module 6000), (Roche Diagnotics GmbH, Mannheim, Germany): LDH [U/L]–lactate dehydrogenase.

The blood biochemical indicators, determined in plasma using a biochemical analyser Roche/Hitachi (Cobas c 501, module 6000), (Roche DiagnosticsGmbH, Mannheim, Germany), were as follows: creatine kinase (CK) [U/l], CK-MB isoenzyme of creatine kinase [U/l], lactate dehydrogenase (LDH) [U/l], total cholesterol [mmol/l], high-density lipoprotein (HDL) [mmol/l], low-density lipoprotein (LDL) [mmol/l], triglycerides [mmol/l], and glucose [mmol/l].

An Alinity I (Abbott Laboratories, IL, USA) immunochemical analyser served to measure the concentrations of testosterone [nmol/l] and TSH [mU/l], and a Cobas e 602 analyser was applied to determine cortisol concentration [µg/dl] (morning hours reference range: 4.82–19.5 µg/dl).

### Statistical analysis

The data were analysed with the Statistica 13.1 software. The statistical significance of the difference between the results obtained before and after CWI was established with the use of Student’s t-test for dependent groups and its non-parametric alternative, the Wilcoxon signed-rank test (for distributions other than normal). The Spearman rank-order correlation test served to analyse the between-parameter correlations. All provided p-values are two-tailed. The statistical significance was set at the value of p ≤ 0.05.

### Institutional review board statement

The study was approved by the Ethics Committee of the Regional Medical Chamber in Krakow, Poland (approval No.: 212/KBL/OIL/2022).

## Results

The participants had a body mass in the range of 69–88 kg and a body height in the range of 170–187 cm. There were no statistically significant changes in blood pressure after CWI as compared with baseline values, while CWI induced a significant reduction of heart rate [28] and body temperature (from 36.45 °C before immersion to a moderate hypothermia of 31.55 °C after immersion; p = 0.003).

In the analysed group (examined before and after CWI), no statistically significant changes were found for the mean values of Na^+^ or K^+^ concentrations (both within standard physiological limits). In turn, Cl^–^ levels were slightly, but significantly decreased after CWI (p ≤ 0.0075 (Table 1).

**Table 1:** Mean values (± standard deviation) of electrolyte concentrations before and after CWI.

Parameter	Reference values	Baseline (n = 12)	After CWI (n = 12)	p (dependent)
Na^+^ [mmol/l]	137 - 147	140.50 ± 1.73	140.33 ± 2.26	0.6886
K^+^ [mmol/l]	3.8 - 5.4	4.59 ± 0.34	4.58 ± 0.42	0.9249
Cl^–^ [mmol/l]	98 - 107	100.86 ± 1.74	100.01 ± 2.14	**0.0075** ^*^

* Significant difference (p < 0.05).

**Fig 1:**
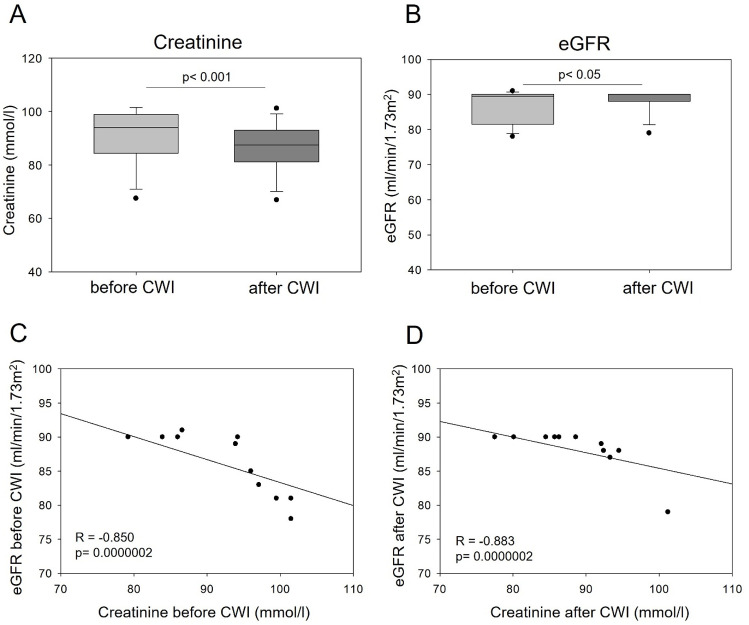
The effect of CWI on (A) creatinine levels, (B) estimated glomerular filtration rate (eGFR) and correlations between these parameters before (C) and after (D) CWI in young healthy males. (A-B) Graphs show the median, 10^th^, 25^th^ 75^th^, and 90^th^ percentile. The statistical differences were calculated using Student’s t-test, n=12. (C-D) Significant negative correlation between creatinine concentration and eGFR both before and after CWI. Spearman rank-order correlation test was used to analyze the correlations.

Statistically significant changes were observed in the mean values of renal profile indicators. The concentrations of urea (p = 0.0019), creatinine (p = 0.0007), and uric acid (p = 0.0293) decreased, while eGFR was significantly increased (p = 0.0425) after CWI (Table 2 and Fig 1, A-B). The levels of renal indicators before and after CWI remained within the standard physiological range. A negative correlation between creatinine levels and eGFR was not significantly affected by CWI (Fig 1, C-D).

**Table 2:** Mean values (± standard deviation) of renal profile parameters before and after CWI.

Parameter	Reference values	Baseline (n = 12)	After CWI (n = 12)	p (dependent)
Urea [mmol/l]	2.15–8.30	5.14 ± 1.07	4.89 ± 0.95	**0.0019** ^*^
Creatinine [µmol/l]	55 - 104	90.57 ± 10.26	86.92 ± 9.07	**0.0007** ^*^
Uric acid [µmol/l]	180 - 420	333.15 ± 54.30	328.61 ± 55.36	**0.0293** ^*^
eGFR [ml/min/1.73 m^2^]	~90–120^**^	86.69 ± 4.46	88.54 ± 3.04	**0.0425** ^*^

* Significant difference (p < 0.05);

** reference values for young male subjects.

eGFR – estimated glomerular filtration rate.

Concerning the indicators of liver profile, statistically significant increases after CWI were observed for total bilirubin (p = 0.0033), AST (p = 0.0023) and ALT (p = 0.0053). Despite the significant increases, the post-CWI values remained within the standard physiological limits. No significant changes in the levels of alkaline phosphatase or GGT were detectable after CWI (Table 3).

**Table 3:** Mean values (± standard deviation) of liver profile parameters before and after CWI.

Parameter	Reference values	Baseline (n = 12)	After CWI (n = 12)	p (dependent)
Total bilirubin [µmol/l]	2 - 21	18.03 ± 12.61	20.05 ± 13.68	**0.0033** ^*^
Alkaline phosphatase [U/l]	40 - 129	67.46 ± 19.09	68.30 ± 19.27	0.3156
AST [U/l]	5 - 38	24.33 ± 5.69	26.66 ± 6.92	**0.0023** ^*^
ALT [U/l]	5 - 41	17.67 ± 4.18	18.50 ± 4.12	**0.0053** ^*^
GGT [U/l]	5 - 55	16.25 ± 3.11	15.25 ± 5.12	0.2143

* Significant difference (p < 0.05)

AST – aspartate transaminase, ALT – alanine transaminase, GGT – gamma-glutamyltransferase.

**Fig 2:**
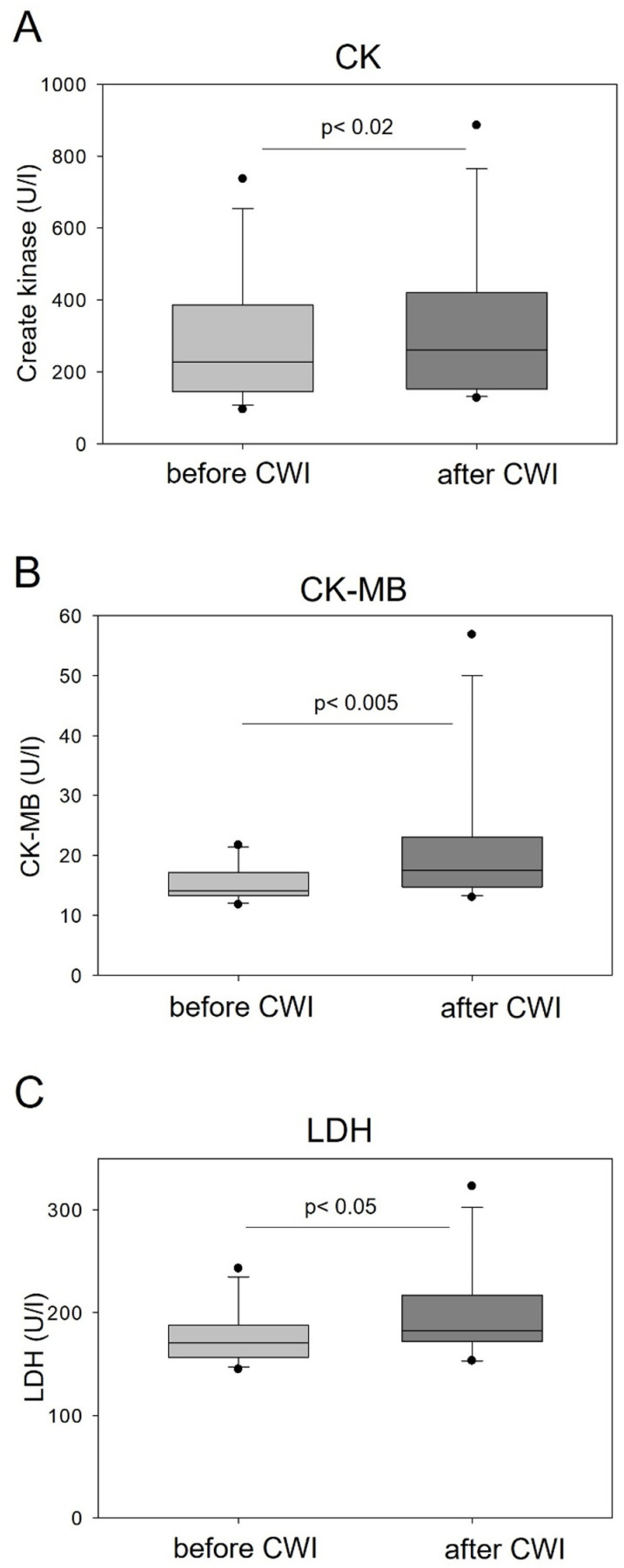
The effect of CWI on blood levels of (A) creatine kinase (CK); (B) CK-muscle brain (CK-MB) and (C) lactate dehydrogenase (LDH) in young healthy males. Graphs show the median, 10^th^, 25^th^ 75^th^, and 90^th^ percentile. The statistical differences were calculated using paired Student’s t-test, n=12.

Both CK (p = 0.0117) and CK-MB (p = 0.0028) values, as well as LDH (p = 0.0336) were significantly increased after CWI, but their values remained mostly within non-pathological limits (Table 4 and Fig 2). Of note, a baseline increase in CK level above 300 U/l was detected in 5 of 12 subjects, whereby in one of them it exceeded the physiological upper limit nearly 4-fold. Even in these volunteers, however, the blood levels of CK were slightly increased by CWI.

**Table 4:** Mean values (± standard deviation) of CK, CK-MB and LDH before and after CWI.

Parameter	Reference values	Baseline (n = 12)	After CWI (n = 12)	p (dependent)
CK [U/l]	24 - 198	284.00 ± 183.14	317.42 ± 214.89	0.0117*
CK-MB [U/l]	4 - 24	15.32 ± 3.04	21.52 ± 12.06	0.0028*
LDH [U/l]	100 - 225	176.72 ± 28.08	199.32 ± 48.37	0.0336*

*Significant difference (p < 0.05)

CK – creatine kinase, CK-MB – isoenzyme of creatine kinase, LDH – lactate dehydrogenase.

With respect to lipid profile parameters in the analysed group examined before and after CWI, a moderate, but statistically significant increase was reported for HDL (p = 0.0270) (within standard limits), with no changes in other indicators (Table 5).

**Table 5:** Mean values (± standard deviation) of lipid profile parameters before and after CWI.

Parameter	Reference values	Baseline (n = 12)	After CWI (n = 12)	p (dependent)
Total cholesterol [mmol/l]	3.0–5.0	3.87 ± 0.70	3.91 ± 0.66	0.4569
HDL [mmol/l]	> 1.0	1.40 ± 0.25	1.46 ± 0.29	0.0270*
LDL [mmol/l]	< 3.0	2.28 ± 0.63	2.33 ± 0.61	0.0526
Triglycerides [mmol/l]	< 1.7	0.84 ± 0.24	0.86 ± 0.24	0.3470

*Significant difference (p < 0.05)

HDL – high-density lipoprotein, LDL – low-density lipoprotein.

There were no statistically significant changes in the concentration of blood glucose, which was 4.77 ± 0.44 at the baseline and 4.93 ± 0.52 after CWI (p = 0.0744).

**Fig 3:**
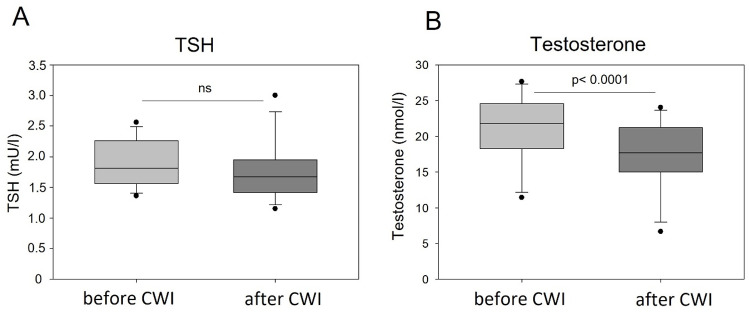
The effect of CWI on blood levels of (A) thyroid-stimulating hormone (TSH) and (B) testosterone in young healthy males. Graphs show the median, 10^th^, 25^th^ 75^th^, and 90^th^ percentile. The statistical differences were calculated using paired Student’s t-test, n=12, ns, not significant.

Furthermore, no statistically significant changes were observed after CWI with regard to the blood hormonal indicators, TSH and cortisol, which decreased slightly after CWI. Notably, the baseline levels of cortisol in blood of the volunteers were relatively high, near the upper reference limit. In contrast to minor changes in TSH and cortisol, a very strong reduction in testosterone concentration was detected upon CWI (p = 0.000037), which was nonetheless still within standard limits (Table 6 and Fig 3).

**Table 6:** Mean values (± standard deviation) of hormonal parameters before and after CWI.

Parameter	Reference values	Baseline (n = 12)	After CWI (n = 12)	p (dependent)
Testosterone [nmol/l]	9.7–32.1	21.22 ± 4.93	17.27 ± 4.89	**0.000037** ^*^
TSH [mU/l]	0.35–4.20	1.89 ± 0.37	1.76 ± 0.48	0.1164
Cortisol [µg/dl]	4.82–19.5	18.91 ± 3.49	17.45 ± 4.17	0.2590

* Significant difference (p < 0.05).

TSH – thyroid-stimulating hormone.

## Discussion

The present study addressed the immediate non-life-threatening physiological responses to cold shock upon immersion in water at a temperature below 4 °C in young males. In the study participants, CWI induced a significant reduction of body temperature from 36.45 °C before immersion to a moderate hypothermia of 31.55 °C after immersion. The results indicate that a single CWI in the described settings decreases Cl^–^ concentration, the values of indicators associated with kidney function, as well as testosterone level within physiological range, whereas a significant increase was observed in the indicators of liver and heart function, and in HDL concentration.

The change in body temperature that occurs as a result of CWI affects all body systems. According to Wittmers [24], CWI affects the central nervous system and respiratory system; it also induces cardiac arrhythmias and vasoconstriction. The cold shock response constitutes the initial sympathetic response to CWI. Liver is among the main organs that warm the blood, i.e., the entire human body. Its function is therefore of critical importance in temperatures below 4 °C. The present study demonstrated a statistically significant increase (within standard limits) of the values of indicators reflecting liver function, i.e., total bilirubin, AST, and ALT. Furthermore, the levels of enzymes associated with heart function, i.e., CK and CK-MB, as well as LDH as a marker of cell death and tissue injuries were significantly increased. Bilirubin increase in blood during a short-term CWI may reflect enhanced red blood cell (RBC) break down. However, in our previous study, no effect of CWI on RBC count was observed [23], which may indicate that cold shock response could reduce bile clearance in healthy subjects. Aminotransferases are involved in protein metabolism and act within the liver, the cardiac muscle, and skeletal muscles. When damage occurs to the cells of these organs, the enzymes are released in large quantities into the circulatory system. Therefore, they are a good indicator of both liver and heart damage. The levels of CK can rise above 4000 U/l after an acute heart muscle ischemia [25], up to 3x10^6^ U/l skeletal muscle injury (rhabdomyolysis), or above 7000 U/l several days after strenuous exercise [26]. In healthy young males, serum CK may be strongly influenced by muscle mass and prior physical exercise [27]. According to a cohort study by Watanabe et al., about 4.3% of middle-aged men have CK levels above 300 U/L [28]. The increased baseline values of CK in our young volunteers may therefore be related to their physical activity in the days prior to the CWI. Apart from CK, which is present in largest amounts in myocardium, skeletal muscle and brain, LDH, which constitutes an important marker of organ failure, was alike consistently increased in the study subjects upon CWI. The exact cellular mechanism behind the elevated serum levels of the abovementioned enzymes is not known, but may be related to cold shock-induced mitochondrial damage, as well as apoptosis of the older cells, including hepatocytes, myocardial and skeletal muscle cells, commonly observed in experimental animals and in vitro studies [29–31]. Notably, the fact that the changes in enzyme levels after CWI remained within standard physiological limits indicates that the rise in their activity was not life-threatening among the study participants and may have contributed to body conditioning.

Regarding the lipid profile, our examinations indicated that CWI slightly increased the concentration of HDL (by 2 mg/dl on average), while the increases in total cholesterol, LDL cholesterol and triglycerides were not statistically significant. This is in line with the results of a study by Ziemann et al. [32] performed among healthy males, in whom the lipid profile improved after whole-body cryostimulation.

The renal function reflected by increased eGFR and reduced blood creatinine level improved after short-term CWI. During CWI, fluid shifts occur between the intravascular and interstitial spaces; fluid loss is also observed, which was manifested by lower Cl^–^ concentrations. The sympathetic nervous system response to cold water can be activated or suppressed very quickly and is dependent on skin temperature [17]. On the other hand, short-term cold stress can cause a diuretic effect and increase eGFR in humans, as cooling of the external body causes peripheral vasoconstriction and a resulting increase in blood flow to the kidneys [33]. Therefore, the observed effect will differ depending on the design of experimental studies, since a prolonged hypothermia was shown to cause kidney injury [34], while the short-term CWI as tested in our study seemed to improve the eGFR without inducing natriuresis. The slightly reduced concentrations of urea and uric acid in blood after CWI remained withing the physiological range in all subjects and most likely did not negatively affect their kidney function.

Testosterone is an anabolic hormone that is responsible for growth and development of tissues, in particular skeletal muscle growth. It is synthesized and secreted by Leyding cells in the testes upon stimulation with luteinizing hormone (LH). According to the previous studies, several minutes of swimming in ice water has no effect on plasma LH [35]. However, a very strong reduction in testosterone concentration was detected in our study upon CWI (p = 0.000037), which was nonetheless still within reference range. No significant changes in other biochemical parameters were observed in the present study. In line with previous reports on cold stress effects in animals or humans [36,37], no statistically significant changes in TSH levels were found in the present study. Cortisol, secreted by the adrenal cortex, also modulates several physiological responses to cold, but in contrast to norepinephrine, tends to decrease after 1-h immersion in cold water [18]. Galbo at al. [38] alike observed that high intensity, short duration bouts of swimming exercise in a cold aquatic environment can result in decreased cortisol levels. In line with this, slight, but not statistically significant decrease in cortisol concentrations was detected after short-term CWI in this study. It must be noted, however, that the pre-CWI cortisol levels were close to the upper reference limits in the study participants, which may indicate that they were strongly stressed by the perspective of cold shock exposure prior to the immersion.

Taken together, temperature shock in cold water induces profound physiological responses, including constriction of blood vessels to maintain internal body temperature, changes in fluid balance, differences in plasma enzyme levels or renal clearance. When a young adult is exposed to temperature shock their body increases heat production through shivering, as increased muscle activity generates heat to maintains vital functions. In our study, young men responded adequately to temperature shock due to their normal thermoregulation, while the risk of pathological findings upon CWI could be higher in other populations, in particular the elderly and the very young who are most sensitive to cold. Furthermore, alcohol consumption dilates skin blood vessels causing increased susceptibility to heat loss even in short-term, whereas a prolonged exposure to cold shock could lead to more extreme and potentially irreversible pathological changes even in organs of healthy subjects. On the other hand, CWI in the above described conditions may contribute to the removal and clearance of old cells in different organs and faster regeneration in athletes, as well as potential conditioning or cold adaptation of young men. The abovementioned results may therefore constitute an essential source of information for winter swimmers and athletes who employ CWI as a post-workout recovery method.

## Conclusions

The study of biochemical blood parameters in healthy males indicate that a short-term immersion in cold water at a temperature below 4 °C, and the air temperature of -15 °C does not constitute a health hazard in young heathy men. The results of the study suggest a rapid response to stress and a positive adaptation of the body to short-term CWI in these subjects. These findings may be of crucial importance to winter swimmers, but also athletes who apply CWI as a recovery method after training.
