# Correction: Single immersion in cold water below 4 °C: A health hazard in young healthy men?

**DOI:** 10.1371/journal.pone.0332529

**Published:** 2025-09-15

**Authors:** Aneta Teległów, Iwona Cicha

There are errors in the Funding statement. The correct Funding statement is as follows: The project was funded within statutory research, project No: 296/BS/INP/2021.

## References

[1] Teległów A, Cicha I. Single immersion in cold water below 4 °C: A health hazard in young healthy men?. PLoS One. 2025;20(5):e0324502. doi: 10.1371/journal.pone.0324502 40408371 PMC12101713

